# Association between chronic low back pain and degree of stress: a nationwide cross-sectional study

**DOI:** 10.1038/s41598-021-94001-1

**Published:** 2021-07-15

**Authors:** Sungwoo Choi, Sangun Nah, Hae-Dong Jang, Ji Eun Moon, Sangsoo Han

**Affiliations:** 1 Department of Emergency Medicine, Soonchunhyang University Bucheon Hospital, 170 Jomaru-ro, Bucheon, 14584 Republic of Korea; 2 Department of Orthopaedic Surgery, Soonchunhyang University Bucheon Hospital, 170 Jomaru-ro, Bucheon, 14584 Republic of Korea; 3 Department of Biostatistics, Clinical Trial Center, Soonchunhyang University Bucheon Hospital, 170 Jomaru-ro, Bucheon, 14584 Republic of Korea

**Keywords:** Health care, Risk factors

## Abstract

Low back pain (LBP) is a very common health problem worldwide, and has a major impact on quality of life. This is a cross-sectional study using data obtained from the Korea National Health and Nutrition Examination Survey (KNHANES) to investigate the health and nutritional status of Korean people, conducted in 2013, 2014, and 2015. The total of 8,473 patients included in the analysis. A 357 (19.34%) subjects in the chronic LBP group and 1,697 (25.61%) subjects in the no chronic LBP group reported no stress (*P* < 0.001). The numbers of subjects reporting mild, moderate, and severe stress in the two groups were 934 (50.6%) vs. 3,785 (57.11%), 432 (23.4%) vs. 910 (13.73%), and 123 (6.66%) and 235 (3.55%), respectively (all *P* < 0.001). Multiple logistic regression analysis with full adjustment for other variables indicated higher OR for severe stress (OR 2.82, *P* < 0.001) than moderate (OR 2.54, *P* < 0.001) and mild (OR 1.55, *P* < 0.001) stress. We confirmed that there was a significant association between chronic LBP and degree of stress. Therefore, the degree of stress should be assessed in clinical treatment of chronic LBP patients.

## Introduction

Low back pain (LBP) is a very common health problem around the world, and it has a direct effect on daily life, causing limitations in activity or problems at work^1,2^. Therefore, there have been many studies regarding LBP. LBP is usually caused by organic problems in anatomical structures, such as bones, intervertebral discs, joints, ligaments, muscles, neural structures, and blood vessels^3,4^. In addition, age, sex, education status, occupational factors, and sitting time have been suggested as risk factors^1,5^. It has also been suggested that psychosocial factors, such as stress, anxiety, and depression, are also associated with LBP^1^. Psychosocial factors were reported to show significant associations with transition from acute to chronic disease^6,7^. In recent years, the degree of stress perceived by individuals in specific occupational groups has been emphasized as a risk factor for chronic LBP^8–10^. In fact, stress is known to have a major effect on health status, affecting cortisol secretion, depression, diabetes mellitus, obesity, and sleep disturbance^10^. However, as there may be inter-individual differences in the degree of actual perceived stress, the physiological stress responses may differ among individuals^11^, so it is necessary to categorize the degree of stress and study the associations with chronic LBP.

Although there have been many studies regarding the association between chronic LBP and stress^7–10^, there have been no reports on the association between chronic LBP and degree of stress in the general population. Therefore, in this study we analyzed the association between chronic LBP and the degree of stress felt by individuals to determine its significance in the general population.

## Methods

### Data collection

Data obtained from the Korea National Health and Nutrition Examination Survey (KNHANES) conducted in 2013 (VI-1), 2014 (VI-2), and 2015 (VI-3) were used. The KNHANES is an annual survey conducted by the Korea Centers for Disease Control and Prevention (KCDC) to investigate the health and nutritional status of Korean people. This survey is conducted by selecting 8000–10,000 individuals from approximately 4000 households through clustered, multistage, stratified, and random sampling. The survey questionnaire consists of three parts, i.e., a health interview using the questionnaire, a health examination, and an interview with experienced medical staff^12^. Participants enrolled in VI-1, 2, and 3 were included in the analysis and, among them, participants aged ≥ 50 years who reported chronic LBP were selected (KNHANES VI-1, 2, and 3 did not survey chronic LBP in participants aged < 50 years). Participants who did not report chronic LBP or stress were excluded.

### Definitions of chronic LBP and degree of stress

Participants who answered “yes” to the question, “Have you had low back pain for more than 30 days in the last 3 months?” were defined as having chronic LBP.

Participants were divided into four categories according to their degree of stress based on their response to the question, “How much stress do you usually feel in your daily life?”: those who responded “very much” were classified as the severe stress group, “a lot” as the moderate stress group, “a little” as the mild stress group, and “hardly any” as the no stress group.

### Demographic and social and health-related variables

Data such as age, sex, height, weight, body mass index (BMI), duration of sleep, smoking status, alcohol consumption, education level, occupation, household income, physical activity, and medical comorbidities were obtained through questionnaires and interviews.

The duration of sleep was evaluated by the question, “How many hours do you usually sleep a day?” participants were classified according to smoking status as non-smokers/ex-smokers or current smokers. Alcohol consumption was categorized as ≤ 1 drink/month, 2–3 drinks/week, and ≥ 4 drinks/week. Education level was divided into four categories according to educational background: elementary school education, middle school education, high school education, and university education. Participants were classified according to occupation as unemployed (student, homemaker, etc.), office work/sales and services, agriculture, forestry, and fishery, machine fitting, and simple labor^13^. Household income was divided into four groups by quartile. Physical activity was defined as moderate intensity aerobic exercise for at least 2 h and 30 min per week and high intensity aerobic exercise for at least 1 h and 15 min per week^4^. Medical comorbidities included in the investigation were hypertension, diabetes mellitus, depression, dyslipidemia, stroke, myocardial infarction, angina, arthritis, asthma, and malignancy.

### Statistical analysis

The general characteristics of the participants were compared between two groups according to the presence or absence of chronic LBP. Student’s *t* test was used for comparisons of continuous variables, and the chi-square test was used for comparisons of categorical variables. Multiple logistic regression was performed to confirm the degree of association between chronic LBP and degree of stress. Odds ratios (ORs) were calculated with 95% confidence intervals (CIs). Multiple logistic regression analysis was performed with three models: Model 1, no adjustments for other variables; Model 2, adjustments for sex and age; and Model 3, adjustments for sex, age, and other environmental factors, such as obesity, sleep duration, smoking, alcohol consumption, education level, household income, occupation, physical activity, and comorbidities. Also, sex-stratified logistic regression analysis was performed. Statistical analyses were performed using IBM SPSS Statistics ver. 26.0 (IBM Corp., Armonk, NY, USA). In all analyses, *P* < 0.05 was taken to indicate statistical significance. In addition, sampling weights were applied to represent the Korean population without bias.

### Ethics declarations

KNHANES VI-1 (approval no. 2013-07CON-03-4C), VI-2 (approval no. 2013-12EXP-03-5C), and VI-3 (approval no. 2015-01-02-6C) were approved by the KCDC Institutional Review Board. All participants voluntarily participated in the study and all provided written informed consent. The study was conducted in accordance with the Declaration of Helsinki, and all research processes were conducted in accordance with the appropriate guidelines and under regulatory supervision.

## Results

**Figure 1:**
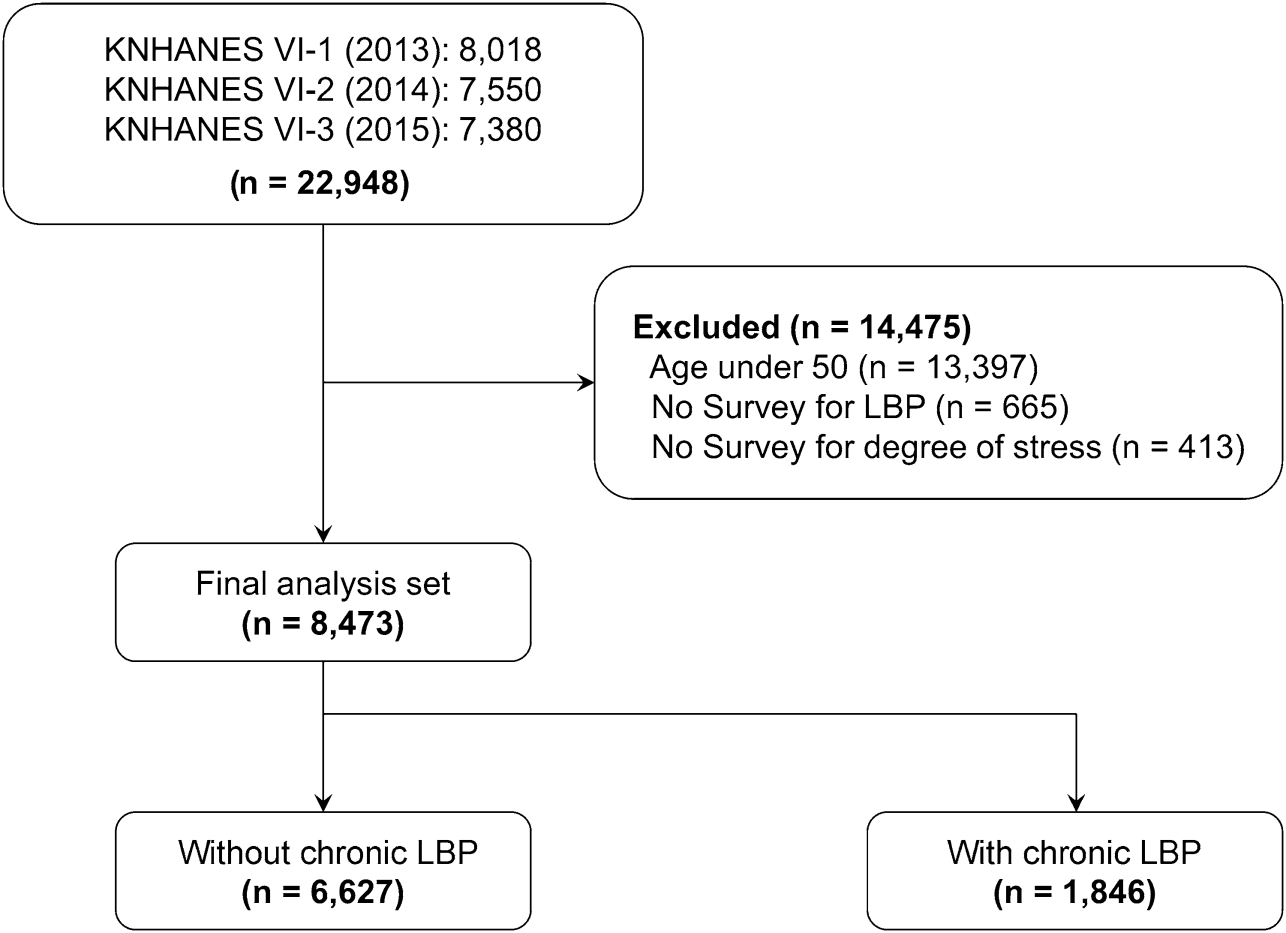
Flow chart of study populations from the 2013–2015 Korea National Health and Nutrition Examination Surveys (KNHANES VI-1, VI-2, and VI-3). *LBP* low back pain.

KNHANES VI-1 (2013), VI-2 (2014), and VI-3 (2015) surveyed 8018, 7550, and 7380 participants, respectively, representing a total study population of 22,948 individuals. Among them, 13,397 participants aged < 50 years, 665 who did not provide responses regarding chronic LBP, and 413 who did not provide responses regarding stress were excluded. The remaining 8473 participants were divided into a no chronic LPB group consisting of 6627 participants (78.21%) and a chronic LPB group consisting of 1846 participants (21.79%) (Fig. 1).

### General characteristics according to presence of chronic LBP

A total of 357 (19.34%) subjects in the chronic LBP group and 1697 (25.61%) subjects in the no chronic LBP group reported no stress (*P* < 0.001). The numbers of subjects reporting mild, moderate, and severe stress in the two groups were 934 (50.6%) vs. 3785 (57.11%), 432 (23.4%) vs. 910 (13.73%), and 123 (6.66%) and 235 (3.55%), respectively (all *P* < 0.001). There were significant differences between the two groups in age, sex, height, weight, BMI, duration of sleep, alcohol consumption, education level, occupation, household income, physical activity, and comorbidities (all *P* < 0.001) (Table 1).

**Table 1:** General characteristics according to chronic LBP.

	Without chronic LBP (*n* = 6627)	With chronic LBP (*n* = 1846)	*P*-value
Age, years	63.19 ± 8.83	67.01 ± 9.01	< 0.001
**Age group, years**			< 0.001
50–59	2,660 (40.14)	476 (25.79)	
60–69	2,217 (33.45)	542 (29.36)	
70–79	1,407 (21.23)	651 (35.27)	
≥ 80	343 (5.18)	177 (9.59)	
**Sex, *n* (%)**			< 0.001
Male	3,126 (47.17)	475 (25.73)	
Female	3,501 (52.83)	1,371 (74.27)	
Height, cm	160.23 ± 8.73	155.84 ± 8.51	< 0.001
Weight, kg	61.94 ± 10.33	59.42 ± 10.07	< 0.001
BMI, kg/m^2^	24.06 ± 3.1	24.41 ± 3.35	< 0.001
**Obesity (BMI), *n* (%)**			0.028
Underweight (< 18.5)	169 (2.55)	49 (2.65)	
Normal (18.5–24.9)	4,102 (61.9)	1,081 (58.56)	
Obese (≥ 25)	2,356 (35.55)	716 (38.78)	
Duration of sleep, h	6.64 ± 1.47	6.38 ± 1.68	< 0.001
**Smoking status, *n* (%)**			< 0.001
Non-smoker/ex-smoker	5,596 (84.44)	1,639 (88.79)	
Current smoker	1031 (15.56)	207 (11.21)	
**Alcohol consumption, *n* (%)**			< 0.001
None	2,476 (37.36)	924 (50.05)	
≤ 1 drink/mo	1,663 (25.09)	461 (24.97)	
2 drinks/mo to 3 drinks/wk	1,918 (28.94)	352 (19.07)	
≥ 4 drinks/wk	570 (8.6)	109 (5.9)	
**Education level, *n* (%)**			< 0.001
Elementary school education	2,464 (37.18)	1,136 (61.54)	
Middle school education	1,242 (18.74)	279 (15.11)	
High school education	1,876 (28.31)	297 (16.09)	
University education	1,045 (15.77)	134 (7.26)	
**Occupation, *n* (%)**			< 0.001
Unemployed (student, homemaker, etc.)	2,998 (45.24)	1,149 (62.24)	
Office work	754 (11.38)	87 (4.71)	
Sales and services	766 (11.56)	164 (8.88)	
Agriculture, forestry, and fishery	1,253 (18.91)	238 (12.89)	
Machine fitting and simple labor	856 (12.92)	208 (11.27)	
**Household income, *n* (%)**			< 0.001
Low	1,702 (25.68)	829 (44.91)	
Low-moderate	1,790 (27.01)	458 (24.81)	
Moderate-high	1,536 (23.18)	294 (15.93)	
High	1,599 (24.13)	265 (14.35)	
**Degree of stress, *n* (%)**			< 0.001
None	1,697 (25.61)	357 (19.34)	
Mild	3,785 (57.11)	934 (50.6)	
Moderate	910 (13.73)	432 (23.4)	
Severe	235 (3.55)	123 (6.66)	
Aerobic physical activity, *n* (%)	1,919 (30.65)	389 (21.19)	< 0.001
**Comorbidities, *n* (%)**			
Hypertension	2,371 (35.78)	858 (46.48)	< 0.001
Diabetes mellitus	889 (13.41)	360 (19.5)	< 0.001
Depression	287 (4.33)	208 (11.27)	< 0.001
Dyslipidemia	1,410 (21.28)	537 (29.09)	< 0.001
Stroke	250 (3.77)	128 (6.93)	< 0.001
Myocardial infarction	97 (1.46)	41 (2.22)	0.0285
Angina	188 (2.84)	105 (5.69)	< 0.001
Arthritis	1,156 (18.41)	783 (42.42)	< 0.001
Asthma	180 (2.72)	128 (6.93)	< 0.001
Malignancy	194 (2.93)	55 (2.98)	0.9688

Values are expressed as the mean ± SD or number (proportion).

*BMI* body mass index, *LBP* low back pain.

*P* < 0.05 was taken to indicate statistical significance.

### Association between chronic LBP and degree of stress

**Figure 2:**
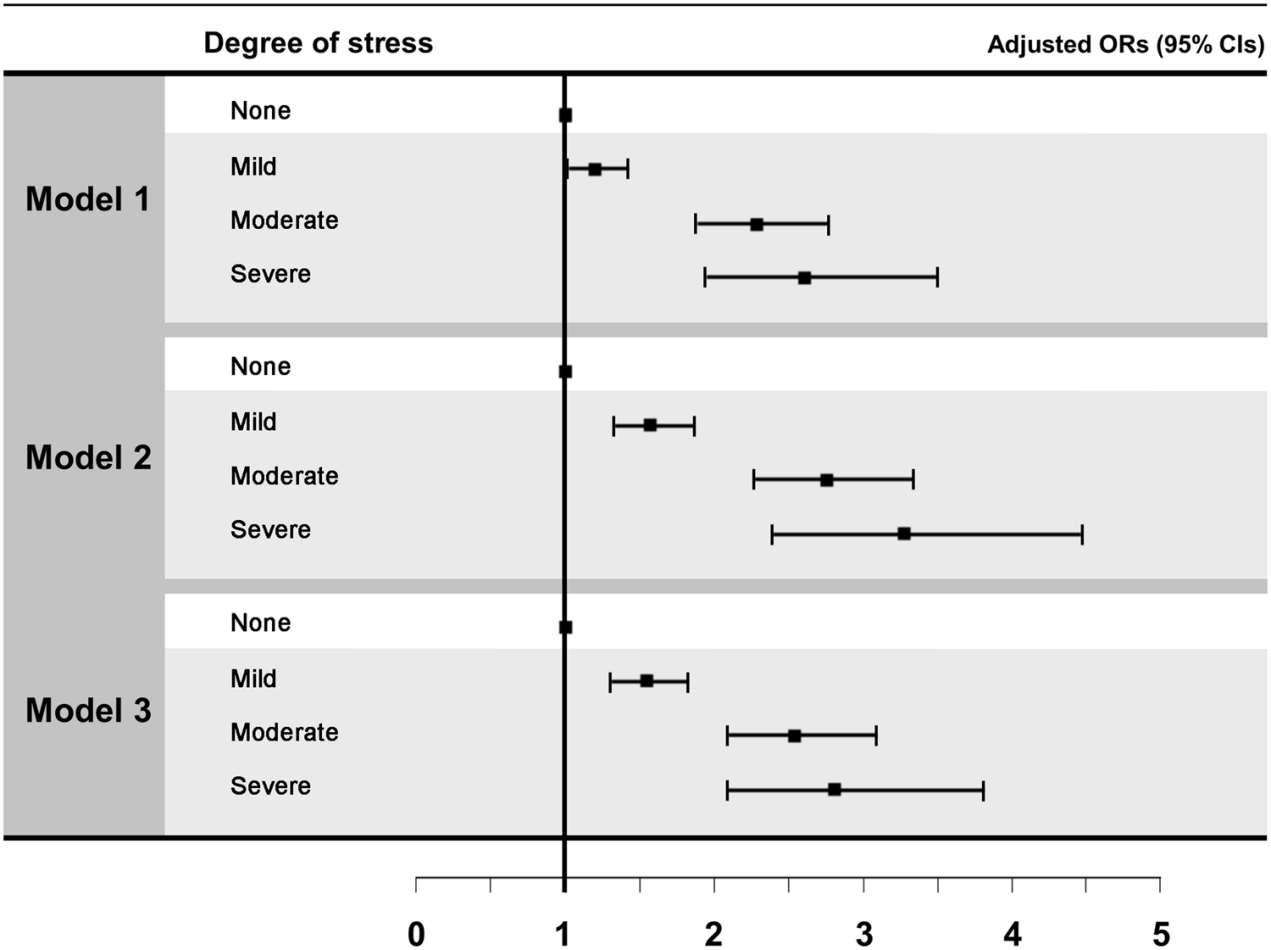
Odds ratio of degree of stress in participants with low back pain. Model 1, no adjustments for other variables; Model 2, adjustments for sex and age; and Model 3, adjustments for sex, age, and other environmental factors, such as obesity, sleep duration, smoking, alcohol consumption, education level, household income, occupation, physical activity, and comorbidities. *CI* confidence interval, *OR* odds ratio.

Multivariate regression analysis was performed to analyze the association between chronic LBP and degree of stress. In Model 1 with no adjustments for other variables, the OR increased from mild stress (OR 1.2, *P* = 0.024) to moderate stress (OR 2.28, *P* < 0.001), and severe stress (OR 2.6, *P* < 0.001). In Model 2 with adjustments for sex and age, the OR increased significantly from mild stress (OR 1.57, *P* < 0.001) to moderate stress (OR 2.74, *P* < 0.001), and severe stress (OR 3.26, *P* < 0.001). Even in Model 3 with adjustments for sex, age, and other environmental factors, such as obesity, sleep duration, smoking, alcohol consumption, education level, household income, occupation, physical activity, and comorbidities, severe stress (OR 2.82, *P* < 0.001) showed a significantly higher OR than moderate stress (OR 2.54, *P* < 0.001) and mild stress (OR 1.55, *P* < 0.001) (Table 2). In addition, the adjusted ORs and 95% CIs of Models 1, 2, and 3 for the association between chronic LBP and degree of stress determined through multiple logistic regression analysis are shown in Fig. 2.

**Table 2:** Association between chronic LBP and degree of stress on multiple logistic regression analysis.

	Model 1			Model 2			Model 3		
	OR	95% CI	*P*-value	OR	95% CI	*P*-value	OR	95% CI	*P*-value
**Degree of stress**									
None	1			1			1		
Mild	1.20	1.03–1.41	0.024	1.57	1.34–1.85	< 0.001	1.55	1.32–1.83	< 0.001
Moderate	2.28	1.89–2.75	< 0.001	2.74	2.27–3.32	< 0.001	2.55	2.1–3.1	< 0.001
Severe	2.60	1.95–3.47	< 0.001	3.26	2.4–4.43	< 0.001	2.83	2.1–3.83	< 0.001

Model 1, no adjustments for other variables; Model 2, adjustments for sex and age; and Model 3, adjustments for sex, age, and other environmental factors, such as obesity, sleep duration, smoking, alcohol consumption, education level, household income, occupation, physical activity, and comorbidities.

*CI* confidence interval, *LBP* low back pain, *OR* odds ratio.

*P* < 0.05 was taken to indicate statistical significance.

In sex-stratified logistic regression analysis, male showed a significantly higher OR than female in all degree of stress; mild stress (OR 1.78 vs. 1.45), moderate stress (OR 3.19 vs. 2.19) and severe stress (OR 3.44 vs. 2.22) (all *P* < 0.001, in the fully adjusted model) (Table 3).

**Table 3:** Sex-stratified logistic regression analysis.

	Unadjusted			Fully adjusted*		
	OR	95% CI	*P*-value	OR	95% CI	*P*-value
**Male**						
None	1			1		
Mild	1.36	1.02–1.81	0.035	1.78	1.33–2.39	< 0.001
Moderate	2.44	1.74–3.42	< 0.001	3.19	2.26–4.52	< 0.001
Severe	3.13	1.85–5.3	< 0.001	3.44	1.95–6.07	< 0.001
**Female**						
None	1			1		
Mild	1.14	0.94–1.38	0.175	1.45	1.20–1.75	< 0.001
Moderate	2	1.59–2.52	< 0.001	2.19	1.73–2.77	< 0.001
Severe	2.16	1.52–3.09	< 0.001	2.22	1.54–3.2	< 0.001

*CI* confidence interval, *LBP* low back pain, *OR* odds ratio.

*Fully adjusted for age, and other environmental factors, such as obesity, sleep duration, smoking, alcohol consumption, education level, household income, occupation, physical activity, and comorbidities.

*P* < 0.05 was taken to indicate statistical significance.

## Discussion

This study, using data obtained through the KNHANES, confirmed the association of chronic LBP with degree of stress in the general Korean population. A total of 22,948 participants were enrolled, among whom 8473 were included in the analysis after excluding those aged < 50 years due to lack of investigation of chronic LBP as well as those who did not provide responses regarding chronic LBP or degree of stress. After adjusting for all factors that may affect chronic LBP, the results of this study confirmed that severity of stress showed a significant positive association with chronic LBP. In particular, severe stress was associated with a 2.8-fold increase in risk of chronic low back pain compared to the general population.

The results of the present study were similar to those of previous research regarding the association between psychological stress and pain^8–11,14^. Most such studies identified associations by sampling from specific occupation groups, such as eldercare, emergency care, and healthcare workers^8–10^. However, differences in occupation may affect the degree of stress, such as job demands, workplace tension, and level of individual control in a specific occupation, and may lead to different rates of work-related injuries and thus affect the occurrence of LBP^15,16^. Therefore, there may be limitations with regard to generalizing the results of these studies to a general population. However, the present study was performed using data from a survey targeting a general population, and not a specific group, with application of sampling weights to obtain representative results. Therefore, our results on the relationship between chronic LBP and degree of stress are more meaningful than those of previous studies in specific limited populations.

Sex is an important factor that can influence both stress and LBP. Multiple factors are suggested to contribute to sex differences in chronic pain, such as variation in stress regulatory systems, sex hormones, genetic factors, and brain structure and function^17^. We performed stratification analysis to find out how much stress contributes to LBP according to sex and, as a result, in men than in women, chronic LBP was more associated with stress.

Stress is a major factor in modulating the pain system through antinociceptive and acute analgesic mechanisms^18^. The stress response is generated through various neurotransmitters (noradrenaline, dopamine, serotonin), peptides (vasopressin), and hormones (cortisol)^19^. Among them, the main components are the hypothalamic–pituitary–adrenal axis (HPA) and the sympathetic nervous system. Triggering of the hypothalamus by stress results in the secretion of corticotrophin-releasing hormone and arginine vasopressin. Adrenocorticotropic hormone is secreted in the posterior pituitary gland and activates noradrenergic neurons in the locus coeruleus/norepinephrine system in the brain. This has a number of consequences, with secretion of many different substances, the most important of which is cortisol, which is regulated through the feedback system of the HPA axis^20^. Chronic stress and repeated high-intensity stress chronically reactivate this stress response, and repeated cortisol surges eventually trigger cortisol dysfunction^21^. As cortisol is a potent anti-inflammatory substance, this dysfunction causes dysfunction of the inflammatory response^22,23^. This can eventually lead to oxidative and nitrosative stress, free radical damage, cellular injury or aging, and systemic tissue degeneration, which can lead to various symptoms, including chronic pain^21,24,25^. These neuroendocrine mechanisms related to stress and pain are consistent with the results of the present study. The results of multiple regression analysis conducted in the present study confirmed that a higher degree of stress is associated with higher OR with chronic LBP, which can be understood based on these mechanisms.

In general, there is a tendency to treat only anatomical or organic problems when evaluating chronic LBP patients in a clinical situation^4^. However, physicians should be aware that the degree of stress is related to chronic LBP. The present study confirmed a significant association between degree of stress and chronic LBP by subjectively expressing the degree of stress according to four categories. Therefore, it is not difficult to evaluate the degree of stress by asking questions in actual clinical situations. In patients complaining about stress, reducing anxiety or fear and increasing physical activity can significantly reduce pain^26^. If the degree of stress is severe or the patient shows depression, antidepressant therapy will be helpful in reducing chronic LBP^27^.

This study had several limitations. First, as this was a cross-sectional study analyzing data obtained from health surveys conducted at the national level, the cause and effect association between chronic LBP and degree of stress could not be determined. However, as the general population was selected by clustered, multistage, and random sampling, both sampling error and selection bias were minimized. Therefore, the association between chronic LBP and degree of stress is highly representative. Second, this was a simple survey on chronic LBP and degree of stress; more detailed evaluations were not performed. Pattern, severity, and duration of pain in chronic LBP were not evaluated, and so could not be quantified. Even with regard to the degree of stress, an objective tool, such as the perceived stress scale, was not used. However, our results indicating a significant association with chronic LBP, based on subjective and simple measurement of the degree of stress, are meaningful. Third, the KNHANES data used in this study were limited to participants aged ≥ 50 years because they did not survey chronic LBP in subjects under 50 years old. Fourth, the influence of ethnicity on the association between degree of stress and chronic LBP cannot be excluded. Therefore, although this study was performed in a general population, further studies in other countries are needed for application of our findings worldwide.

## Conclusion

This cross-sectional study, conducted using national health survey data obtained at a national level, confirmed a significant direct association between degree of stress and chronic LBP. Therefore, it is necessary to recognize that degree of stress and chronic LBP are related, and clinicians should evaluate the degree of stress when treating patients with chronic LBP.

## Data Availability

The dataset of this study and the KNHANES data are available through the Korea Centers for Disease Control and Prevention website (https://knhanes.cdc.go.kr/).

## References

[1] Hoy D, Brooks P, Blyth F, Buchbinder R (2010). The epidemiology of low back pain. Best Pract. Res. Clin. Rheumatol..

[2] Lidgren L (2003). The bone and joint decade 2000–2010. Bull World Health Organ..

[3] Deyo RA, Weinstein JN (2001). Low back pain. N. Engl. J. Med..

[4] Park, S.-M. *et al*. Depression is closely associated with chronic low back pain in patients over 50 years of age: A cross-sectional study using the sixth Korea National Health and Nutrition Examination Survey (KNHANES VI-2). *Spine (Phila Pa 1976).* **43**(18), 1281–1288 (2018). 10.1097/BRS.0000000000002595 29462063

[5] Park S-M (2018). Longer sitting time and low physical activity are closely associated with chronic low back pain in population over 50 years of age: a cross-sectional study using the sixth Korea National Health and Nutrition Examination Survey. Spine J..

[6] Linton SJ. A review of psychological risk factors in back and neck pain. *Spine (Phila Pa 1976)* **25**(9), 1148–1156 (2000). 10.1097/00007632-200005010-00017 10788861

[7] Pincus, T., Burton, A.K., Vogel, S., Field, A.P. A systematic review of psychological factors as predictors of chronicity/disability in prospective cohorts of low back pain. *Spine (Phila Pa 1976)* **27**(5), E109–E120 (2002). 10.1097/00007632-200203010-00017 11880847

[8] Tsuboi Y, Ueda Y, Naruse F, Ono R (2017). The association between perceived stress and low back pain among eldercare workers in Japan. J. Occup. Environ. Med..

[9] Kılınç A, Çalışkan Pala S, Arslantaş D, Ünsal A (2020). The evaluation of low back pain and perceived stress among prehospital emergency care workers. Eur. J. Public Health..

[10] Vinstrup J, Jakobsen MD, Andersen LL (2020). Perceived stress and low-Back pain among healthcare workers: A multi-center prospective cohort study. Front Public Health..

[11] Vachon-Presseau E (2018). Effects of stress on the corticolimbic system: Implications for chronic pain. Prog. Neuropsychopharmacol. Biol. Psychiatry..

[12] Kweon S (2014). Data resource profile: The Korea national health and nutrition examination survey (KNHANES). Int. J. Epidemiol..

[13] Punnett L (2005). Estimating the global burden of low back pain attributable to combined occupational exposures. Am. J. Ind. Med..

[14] Arguelles LM (2006). A twin study of posttraumatic stress disorder symptoms and chronic widespread pain. Pain.

[15] Adriaenssens J, Hamelink A, Bogaert PV (2017). Predictors of occupational stress and well-being in first-line nurse managers: A cross-sectional survey study. Int. J. Nurs. Stud..

[16] Zadow AJ, Dollard MF, McLinton SS, Lawrence P, Tuckey MR (2017). Psychosocial safety climate, emotional exhaustion, and work injuries in healthcare workplaces. Stress Health..

[17] Bartley, E.J., Roger, B.F. Sex differences in pain and stress. in *Neuroscience of Pain, Stress, and Emotion* (ed. Bartley, E.) 87–89 (Academic Press, 2016)

[18] Butler RK, Finn DP (2009). Stress-induced analgesia. Prog. Neurobiol..

[19] McEwen BS (1998). Stress, adaptation, and disease: Allostasis and allostatic load. Ann. N. Y. Acad. Sci..

[20] Guilliams TG, Edwards L (2010). Chronic stress and the HPA axis. Standard..

[21] Hannibal KE, Bishop MD (2014). Chronic stress, cortisol dysfunction, and pain: A psychoneuroendocrine rationale for stress management in pain rehabilitation. Phys. Ther..

[22] Tsigos, C., Chrousos, G.P. Hypothalamic–pituitary–adrenal axis, neuroendocrine factors and stress. *J. Psychosom. Res. Z* **53**(4), 865–871 (2002). 10.1016/s0022-3999(02)00429-4 12377295

[23] Fries E, Hesse J, Hellhammer J, Hellhammer DH (2005). A new view on hypocortisolism. Psychoneuroendocrinology.

[24] Maes M, Galecki P, Chang YS, Berk M (2011). A review on the oxidative and nitrosative stress (O&NS) pathways in major depression and their possible contribution to the (neuro) degenerative processes in that illness. Prog. Neuropsychopharmacol. Biol. Psychiatry..

[25] Zunszain PA, Anacker C, Cattaneo A, Carvalho LA, Pariante CM (2011). Glucocorticoids, cytokines and brain abnormalities in depression. Prog. Neuropsychopharmacol. Biol. Psychiatry..

[26] Indahl, A., Velund, L., Reikeraas, O. Good prognosis for low back pain when left untampered. A randomized clinical trial. *Spine (Phila Pa 1976)* **20**, 473–477 (1995). 10.1097/00007632-199502001-00011 7747232

[27] Chan HN, Fam J, Ng B-Y (2009). Use of antidepressants in the treatment of chronic pain. Ann. Acad. Med. Singap..

